# Mucormycosis in Burn Patients

**DOI:** 10.3390/jof5010025

**Published:** 2019-03-21

**Authors:** Pauline Devauchelle, Mathieu Jeanne, Emilie Fréalle

**Affiliations:** 1 CHU Lille, Centre des Brûlés, F-59000 Lille, France; pauline.clementgrandcourt@chru-lille.fr (P.D.); mathieu.jeanne@chru-lille.fr (M.J.); 2 Univ. Lille, Inserm, CHU Lille, CIC 1403-Centre d’Investigation Clinique, F-59000 Lille, France; 3 CHU Lille, Laboratoire de Parasitologie-Mycologie, F-59000 Lille, France; 4 Univ. Lille, CNRS, Inserm, CHU Lille, Institut Pasteur de Lille, U1019–UMR8204-CIIL-Center for Infection and Immunity of Lille, F-59000 Lille, France

**Keywords:** mucormycosis, burn, cutaneous, qPCR, outbreak, molecular typing, environmental source, nosocomial, *Saksenaea*, Apophysomyces

## Abstract

Patients with extensive burns are an important group at risk for cutaneous mucormycosis. This study aimed to perform a systematic review of all reported mucormycosis cases in burn patients from 1990 onward. A Medline search yielded identification of 7 case series, 3 outbreaks, and 25 individual cases reports. The prevalence reached 0.04%–0.6%. The median age was 42–48 in the case series and outbreaks, except for the studies from military centers (23.5–32.5) and in individual reports (29.5). The median total body surface area reached 42.5%–65%. Various skin lesions were described, none being pathognomonic: the diagnosis was mainly reached because of extensive necrotic lesions sometimes associated with sepsis. Most patients were treated with systemic amphotericin B or liposomal amphotericin B, and all underwent debridement and/or amputation. Mortality reached 33%–100% in the case series, 29%–62% during outbreaks, and 40% in individual cases. Most patients were diagnosed using histopathology and/or culture. Mucorales qPCR showed detection of circulating DNA 2–24 days before the standard diagnosis. Species included the main clinically relevant mucorales (i.e., *Mucor*, *Rhizopus*, *Absidia/Lichtheimia*, *Rhizomucor*) but also more uncommon mucorales such as *Saksenaea* or *Apophysomyces*. Contact with soil was reported in most individual cases. Bandages were identified as the source of contamination in two nosocomial outbreaks.

## 1. Introduction

Patients with extensive burns are an important group at risk for cutaneous mucormycosis. The first two cases were reported in 1961 by Rabin et al. [1]. Several reviews on cutaneous mucormycosis have been published, showing mostly localized infection or accompanied with deep extension, but few have shown dissemination [2]. Clinical presentation is characterized by rapid progression to necrosis and a better prognosis than other clinical forms of mucormycosis (31% mortality). Although these reviews include burn patients, none is specifically focused on them. In 2005, Roden et al. identified 11 cases of cutaneous mucormycosis in burns among 176 patients (6%) before 2004 [2]. Then, Ledgard et al. reviewed all published cutaneous mucormycosis cases from 1966 to 2006 and found that burns were amongst the most commonly reported factors, representing 19 out of 145 cases (13%) [3]. Lastly, in 2009, Skiada and Petrikkos analyzed seven case reports published from 2004 and 2008 [4], and, in 2012, Skiada et al., identified two supplementary cases in 2009–2010 [5].

Since no review focusing on burn patients has been published so far, this study aimed to perform a systematic review of all reported mucormycosis cases in burn patients. We undertook a Medline search using the keywords “mucormycosis” or “*Lichtheimia*” or “*Absidia*” or “*Mucor*” or “*Rhizopus*” or “*Rhizomucor*” and “burn”. Non-English language papers were excluded. We included individual case reports (including those which had been analyzed in previous reviews on cutaneous mucormycosis), case series reports (i.e., including at least two patients), and outbreak reports published from 1990 onward, in order to collect data on the prevalence of mucormycosis in burn patients; clinical, diagnosis, and therapeutic features; the species involved; and the results of environmental investigations in individual cases and outbreaks. Recent data on molecular diagnosis (real-time PCR) were also included.

## 2. Results of PubMed Search

A Medline search yielded seven case series from the USA (*n* = 3), Australia (*n* = 1), France (*n* = 1), Greece (*n* = 1), and across Europe (*n* = 1) [6,7,8,9,10,11,12]. Three nosocomial outbreaks were reported: one in Belgium in 2005 and two in France in 2018 [13,14,15,16]. Lastly, 25 reports of individual cases were identified between 1990 and 2019, including publications from the USA (*n* = 12, with three cases occurring in the Middle East (Afghanistan, Iraq) and one in Japan) [17,18,19,20,21,22,23,24,25,26,27,28], from Asia (*n* = 6; two in China, three in India, one in Iran) [29,30,31,32,33,34], from Europe (*n* = 3; in Czech Republic, Germany, and the U.K.) [35,36,37], from Australia (*n* = 3) [3,38,39], and from Ecuador (*n* = 1) [40]. The data from case series and outbreaks are summarized in Table 1. The epidemiological, clinical, diagnosis, and therapeutic features of burn patients with mucormycosis from individual case reports are presented in Table 2.

## 3. Prevalence of Mucormycosis in Burn Patients

The prevalence of mucormycosis was reported from three cases series between 1991 and 2009 in the Brooke Army Medical Center (USA), ranging from 0.1% to 0.6% [6,7,8]. Similar prevalences of 0.5% and 0.6% have been more recently reported in France and in Greece, in studies covering the 2000–2011 and 2005–2014 periods, respectively [9,10]. Katz et al. reported a lower prevalence of 0.04% in a study conducted between 2001 and 2011 in Australia [11]. Increased prevalences of mucormycosis were observed during two outbreaks in Belgium (18.5%) [13] and in France (10.7%) [16]. Two case series and one outbreak report have differentiated colonization by mucorales, which, according to the system developed at the United States Army Institute of Surgical Research Burn Center, is defined by fungal presence without penetration into viable tissue (superficial colonization, microorganisms in non-viable tissue or microorganisms at the interface of viable tissue), and infection, which corresponds to an invasion of fungi into viable tissue below the eschar of a specimen (microinvasion of viable tissue, deep or generalized invasion of viable tissue, or microvascular invasion) [41]. In these three studies, colonized patients represented 29% to 41% of patients with positive culture [8,10,13].

## 4. Epidemiological, Clinical, and Therapeutic Features of Mucormycosis in Burn Patients

In case series and outbreaks, the median age ranged from 42 to 48, except for in studies from military centers in the USA or in France, which reported lower median ages of 23.5 and 32.5, respectively (Table 1). When analyzing data from the 25 individual cases retrieved using Medline, we found a median age of 29.5 (10–63), which was lower than that in case series and outbreaks (Table 2). Cases mostly occurred in men in the case series (6 men vs. no women) and individual reports (18 men vs. 6 women), but the number of men and women who developed mucormycosis was similar during outbreaks (Table 1). The median percentage of the total body surface area (TBSA) affected by a burn reached 42.5% to 65% in case series and outbreaks (Table 1) and 58% (4%–96%) when analyzing individual case reports (Table 2). Most of the patients with lower TBSA (<40%) presented with diabetes mellitus, which was the most common underlying condition (20%). Hyperglycemia poorly controllable by high-dose insulin was reported prior to the diagnosis of mucormycosis in two patients with diabetes [17,29]. It was associated with metabolic acidosis in one patient [17]. Few patients had other underlying conditions, such as ethanol or drug abuse or cardiovascular diseases. The most common clinical forms of mucormycosis were cutaneous, but three patients presented rhinocerebral, cerebral, or retro-orbitary infection (12%) (Table 2). Pulmonary localization was reported in one patient during an outbreak [14], and chronic osteomyelitis of the lower limb was associated with sinus infection 5 months after a burn in one patient [33]. One study reported septic shock and uncontrollable disseminated intravascular coagulation causing fatal cerebrovascular stroke [35]. From a clinical point of view, an invasive mucormycosis infection cannot be related to any specific cutaneous lesion. The literature describes various skin lesions, none being pathognomonic [12]: in most case reports, the diagnosis is reached because of extensive necrotic lesions sometimes associated with sepsis, in most cases after a first excision procedure [3,11,19,20,29,30,32,35,37]. In the other case reports, cutaneous lesions are not described, and the diagnosis is made based on an early skin biopsy [12,23,26].

Most patients were treated with systemic amphotericin B or liposomal amphotericin B, and all underwent debridement and/or amputation (Table 2). Local treatment with amphotericin B was used in three patients. Although posaconazole and isavuconazole can also be considered for the treatment of mucormycosis [42,43], only three reports of treatment with posaconazole and/or isavuconazole were identified in three patients, including two patients who underwent initial treatment with liposomal amphotericin B [14,33]. In the third patient, posaconazole was followed by isavuconazole and associated with local amphotericin B [27]. Surprisingly, one patient with low TBSA (4%–5%) who presented diabetes mellitus with hyperglycemia and was poorly controllable by high-dose insulin was successfully treated with voriconazole, which is known to lack meaningful in vitro activity against Mucorales [29]. Mortality ranged from 33% to 100% in the case series and from 29% to 62% during outbreaks. The overall mortality in the individual cases was 40%, with a 100% mortality rate in the three patients diagnosed with rhinocerebral, cerebral, or retro-orbitary infection (Table 2).

## 5. Mycological Diagnosis

### 5.1. Standard Diagnosis

Diagnosis of mucormycosis in burn patients generally relies on the histopathological analysis and culture of skin biopsies. However, since fungal colonization may represent 29% to 41% of patients with detection of mucorales in culture (Table 1), isolation alone may not be helpful to differentiate colonization and infection. When analyzing the individual reports in Table 2, most patients were found to have been diagnosed using histopathology (61%) and/or culture (87%). No antifungal susceptibility testing was performed. The median delay between burn and diagnosis was 13 days (3–150 days), and in 14 out of 21 patients (67%), mucormycosis manifested within the initial 2 weeks after admission. This short delay was consistent with the inoculation of the patient’s burn wounds with the fungi during the burn injury, whereas the 36 day median delay between admission and diagnosis (18–95 days) reported in one outbreak was more indicative of a nosocomial contamination [14].

### 5.2. Molecular Diagnosis

Several real-time PCR methods have been developed to detect and identify Mucorales in tissue samples. Most of them target clinically relevant mucormycetes of the Mucorales order, including *Mucor, Rhizopus*, *Rhizomucor*, and *Absidia/Lichtheimia* species [44,45]. Interestingly, a real-time PCR targeting *Lichtheimia*, *Rhizopus/Mucor*, and *Rhizomucor* for detection of mucorales DNA in serum developed by Millon et al. [46,47] was retrospectively and prospectively evaluated in 75 burn patients during an outbreak in Saint-Louis hospital. This study showed detection of circulating mucorales DNA 2 to 24 days before the standard diagnosis (direct examination or culture) in 10 patients with mucormycosis (including 2 patients from two other centers) and a decline of circulating mucorales DNA in patients under treatment [16]. Furthermore, a persistent positive qPCR after treatment initiation was shown to be associated with death.

### 5.3. Species Involved

Mucorales species identified in infected or colonized patients from the case series included mainly *Mucor* spp. (*n* = 11) (Table 1). *Rhizopus* spp., *Absidia/Lichtheimia* spp., and *Rhizomucor* spp. were identified in four, two, and two patients, respectively. Rare species such as *Saksenaea vasiformis* or *Saksenaea erythrospora* were isolated in two patients that were injured in Iraq or Afghanistan. Two out of the three reported outbreaks were due to *Absidia/Lichtheimia* spp. In the third one, *M. circinelloides* was the main species (in six out of eight cases). In individual case reports, mucorales species were isolated by culture in 16 patients. *Saksenaea* species were the most frequent species (*n* = 4; in Iraq, Afghanistan, Australia, and Ecuador), followed by *Absidia/Lichtheimia* spp. (*n* = 6; in Afghanistan, U.K., USA, Czech Republic, and India (*n* = 2)), *Mucor* spp. (*n* = 3: in the USA), *Rhizopus* spp. (*n* = 3; in the USA, China, Germany), and *Apophysomyces* spp. (*n* = 2; in Australia, Japan). *Rhizomucor pusillus* was identified in one patient (Australia), and *Cunninghamella elegans* in another patient (Afghanistan). For three patients, the mucorales species was not identified.

Morphological identification was confirmed by molecular methods only in four patients with *Saksenaea* spp. or *Apophysomyces variabilis* infection, including one patient who had coinfection with *S. vasiformis*, *C. elegans*, and *Lichtheimia corymbifera* (Table 2). Identification was also confirmed in two out of three outbreaks, yielding *M. circinelloides* or *L. corymbifera* [16] and *L. ramosa* or *L. ornata* [14], but no molecular identification was reported in the case series.

Mixed infections due to two distinct mucorales or one mucorale and another filamentous fungus are reported in a few studies. For example, a combined infection by *Absidia/Lichtheimia* sp., *Aspergillus fumigatus*, and *Fusarium* sp. was reported by Lipovy et al. [36]. Schaal et al. also found two mucormycosis cases with co-infection with *Aspergillus* [10], and Atty et al. and Schofield et al. found other co-infections with *Fusarium* sp. [6,19]. Lastly, a co-infection with several mucorales species was reported by Farmer et al. (*S. vasiformis*, *C. elegans*, and *L. corymbifera* associated with *Aspergillus*, *Geotrichum*, *Alternaria*) and by Fréalle et al. (*L. ornata* and *Rhizopus delemar*) [14].

Bacterial co-infections or antibiotic treatments, which put burn patients at particular risk of invasive fungal infection, were mentioned in 14 individual case reports. The higher mortality in these patients (57%) than in patients with no bacterial infection or antibiotic treatment (18%) was consistent with increased disease severity if there are confirmed or suspected bacterial co-infections (Table 2).

## 6. Environmental Investigations of Mucormycosis in Individual Cases and Outbreaks

When analyzing the circumstances of burns in individual reports, contact with soil after the burn injury was reported in four cases, with the patient rolling in soil or muddy ground water to extinguish the flames, and was consistent with an inoculation with the fungi at the time of the burn injury (Table 2). Burn causes included traffic accident (*n* = 4), airplane crash (*n* = 2), improvised explosive device blast (*n* = 3) or workplace explosion (*n* = 1), gasoline fire (*n* = 3), domestic accident (with tap water or during cooking; *n* = 3), and high-voltage electric contact (*n* = 1). The absence of exposure to an infectious environment was specified in one report [35]. In the case series, combat-related burns [8,10] or forest blaze [9] were reported. Altogether, these etiologies included common locations/settings in which the burns occur, such as cooking, tap water, or electric contact [48], but common etiologies for flames in the household, such as barbecues or the use of gasoline, were not reported. Conversely, trauma circumstances were over-represented.

Environmental investigations were performed in the three outbreaks of mucormycosis that were reported in burn units [13,14,15,16]. During the first outbreak, which was due to *L. corymbifera* and reported in 2005 in Belgium [13], air, surface, or water samples and non-sterile Elastoplast bandages were analyzed by culture. These analyses yielded detection of *L. corymbifera* in bandages, confirming that Elastoplast bandages were the source of contamination of patients. However, no molecular confirmation was performed. Bandages were also identified as the potential source of contamination in seven serial cases of *Lichtheimia* mucormycosis in the burn unit of the University Hospital of Lille between 2013 and 2016 [14]. Analysis by culture of non-sterile crepe and Elastoplast bandages showed only one crepe bandage positive for *Lichtheimia* sp., but the genotypic relatedness of the strains between patients and bandage strains could not be confirmed since further multi-locus sequence typing (MLST) of this environmental strain and of the seven *Lichtheimia* clinical strains revealed different species in patients (*L. ramosa* and *L. ornata*), and in the bandage (*L. corymbifera*). However, *Lichtheimia* spp. and *Lichtheimia* species-specific qPCR revealed the presence of *L. ramosa* and/or *L. ornata* in most crepe and elasticized bandages, which were frequently contaminated by different *Lichtheimia* strains. A third outbreak of mucormycosis due to *M. circinelloides f. circinelloides* was reported in the burn units of the Saint-Louis Hospital, Paris, France, and another in hospital in a Paris suburb (Hôpital d’Instruction des Armées, Clamart, France) during the same period; these occurred between 2013 and 2015 and involved six and four patients, respectively [15,16]. MLST and whole-genome sequencing analysis of the patient’s isolates revealed a large diversity of isolates in the different patients, with most strains being patient-specific and within the same patient, with two patients being infected by a mixture of strains. These data were consistent with the presence of a local environmental “reservoir” containing clonally unrelated isolates (probably linens or Elastoplast). However, this reservoir could not be identified since all the environmental samples that were analyzed by culture were negative, and, although *Mucor*/*Rhizopus* qPCR was also used, it was positive only in the Bair Hugger filters that were used during the hospitalization of three patients.

## 7. Conclusions

Mucormycosis is a rare disease in burn patients but remains difficult to treat and is often lethal. The clinical and prognostic features are similar to those in other patients with cutaneous mucormycosis (such as trauma or surgery). The treatment is also similar, relying on early diagnosis; extensive surgical debridement (including amputation when appropriate), which is rendered necessary for the complete resection of necrotized and infected tissue; and intravenous administration of high doses of amphotericin B or liposomal amphotericin B [4]. Glucose control and correction of acidosis are also required in patients with diabetes mellitus. qPCR detection of mucorales DNA in serum represents a promising tool to improve outcomes for patients by enabling early diagnosis. It could also be useful to differentiate colonization versus infection. The species responsible for mucormycosis in burn patients include the main clinically relevant mucorales (i.e., *Mucor Rhizopus*, *Absidia/Lichtheimia*, *Rhizomucor*) but also uncommon mucorales such as *Saksenaea* or *Apophysomyces*. Nevertheless, data on molecular identification would be needed to attain reliable epidemiological data. Since inoculation of soil-dwelling moulds into wounds can occur at the time of injury, the context in which burns occur needs to be taken into account in order to evaluate the risk of mucormycosis. In nosocomial outbreaks, bandages appeared to be the main source of contamination with various mucorales strains, underlying the need to use sterile bandages on burn patients.

## Figures and Tables

**Table 1:** Case series or outbreaks of mucormycosis in burn patients.

	Country	Type of Unit	Period of Study	Number of Patients Admitted	Number of Cases	Median Age; Gender	Median TBSA (%)	Mucorales Species	Prevalence of Patients with Mucormycosis	Mortality	Refs
Case Series	USA (Texas)	Military adult burn center	1991–2002	2651	16 cases,11 colonized patients	NA	NA	NA	0.6% (cases), 1.0% (including colonization)	NA	[7]
			Apr 2000–March 2005	2036	2	NA	NA	*Mucor* sp. (2)	0.1%	100%	[6]
			Jan 2003–Nov 2009	2449	12 (7 with positive culture)	23.5; NA	60	*Mucor circinelloides* (2), *Saksenaea vasiformis* (1), *Saksenaea erythrospora* (1), *Pythium aphanidermatum* * (1)	0.5%	54%	[8]
	Australia (Melbourne)	Adult Burns Service	Jan 2001–June 2011	NA	3	42; 3M/0F	65	*Mucor* sp. (1), *Mucor ramosissimus* (1), *Absidia corymbifera* (1)	0.04%	33%	[11]
	France (Paris)	Military adult burn center	2000–2011	1849	6 cases, 3 colonized patients	32.5; NA	42.5	*Mucor* sp. (2), *Mucor circinelloides* (2), *Mucor racemosus* (1), *Lichtheimia ornata* (1), *Rhizopus oryzae* (1), *Rhizomucor variabilis* ^£^ (1), *Rhizopus stolonifer* (1)	0.3 % (cases), 0.5% (including colonization)	33%	[10]
	Europe	NA	2005–2007	NA	7	NA	NA	NA	NA	33%	[12]
	Greece (Athens)	Adult Burns Service	2005–2014	477	3	47; 3M/0F	50	*Rhizomucor* (2), *Rhizopus* (1)	0.6%	67%	[9]
Outbreaks	Belgium (Liège)	Plastic Surgery and burns unit	May–Sept 2004	27	5 cases, 2 colonized patients	NA	NA	*Absidia corymbifera* (7)	18.5 % (cases), 25.9% (including colonization)	60%	[13]
	France (Paris)	Burn intensive care unit	Oct 2013–Feb 2016	75	8	48; 4M/4F	62	*Mucor circinelloides* (6), *Mucor* or *Rhizopus* (1), *Lichtheimia corymbifera* (1)	10.7%	62%	[15,16]
	France (Lille)	Burn unit	Nov 2013–July 2016	NA	7	43; 4M/3F	50	*Lichtheimia ramosa* (5), *Lichtheimia ornata* (1), *Lichtheimia ornata/Rhizopus delemar* (1)	NA	29%	[14]

NA = Not available; M = Male, F = Female; * *Pythium aphanidermatum*, which belongs to the Oomycota, was erroneously included in the mucormycosis cases in the study by Mitchell et al. Median age, total body surface area (TBSA), prevalence, and mortality are given for the 11 mucormycosis cases and the *P. aphanidermatum* case; ^£^ *Rhizomucor variabilis* var. *variabilis* is currently known as *Mucor irregularis*.

**Table 2:** Epidemiological, clinical, diagnosis, and therapeutic features of burn patients with mucormycosis from individual cases or case series reports.

Country, Year of Publication, and Reference	Age/ Sex	Circumstances of Burn	TBSA	Underlying Condition *	Localization	Clinical Presentation	Date of Diagnosis and Method (H = Histology, M = Microscopy, C = Culture)	Species and Date of Isolation (** = Molecular Confirmation of Identification)	Antifungal (Date of Beginning) and Other Treatments	Outcome
Australia (Darwin) 1990 [38]	45/M	Covered with moist soil during his attempts to extinguish the flames	25	No	Right ankle	Fever, subcutaneous swelling	D32 (M,C)	*Apophysomyces elegans*	AmB IV (D33) Debridement Amputation	Improved
USA (Washington) 1993 [24]	42/F	Doused in alcohol and ignited. Rolling in mud to extinguish the flames	67	Ethanol abuse *Bact & ABT*	Upper extremities	Edema of forearm, necrosis of muscles of flexor compartment, secondary necrosis of left hand	D12 (C) & D16 (H,C)	*Mucor*	AmB IV (D17) Debridement Amputation right arm and left hand	Deceased D22
USA (Durham) 1997 [25]	28/F	Tap water	NA	Diabetes mellitus	Right hand and forearm	Life-threatening necrosis of hand and forearm	NA (H,C)	*Rhizopus oryzae*	Arm amputation	Improved
China (Dalian) 1998 [32]	40/F	Liquid gas explosion	85	No	Trunk and limbs	Fever, tachycardia, extensive edema, multifocal indurated nodules rapidly extensive	D6 (H,C)	*Rhizopus rhizopodiformis*	AmB IV, oral 5-FC (D7) AmB local (D10) Debridement,	Improved
USA (Ohio) 1999 [17]	62/M	Airplane crash	29	Diabetes mellitus, hypertension, coronary disease	Rhinocerebral	Depression of the right supraorbital rim, multiple organ failure	D4 (H)	NA	Debridement	Deceased D11
Ecuador (Guayaquil) 2006 [40]	13/M	Domestic accident. Patient rolled on the ground to extinguish the flames then applied oil to prevent water or plasma loss.	65	No *Bact & ABT*	NA	Lysis of an area of the graft in the sacrum region with fetid and purulent exudate	D7 (M,C)	*Saksenaea vasiformis*	AmB IV (D8) Debridement	Improved
Australia (Adelaide) 2008 [3]	35/M	Motor vehicle accident with car burning. Rolling in dirt to extinguish the flames.	60	No *Bact & ABT*	Arms, legs	Severe aggressive and rapid necrosis of right lower leg, new necrotic areas in previously debrided zones	D5 & D17 (C)	*Saskena vasiformis* **	L-AmB IV (D19) Debridement Below knee and above elbow amputation	Improved
U.K. (Birmingham) 2008 [37]	27/M	Road traffic accident in Kenya	45	No *Bact & ABT*	Trunk, arms, legs, face	Circular black area in the burned zone	D21 (H,C)	*Absidia corymbifera*	L-AmB IV (D21) Local nystatin (D25) Debridement	Improved
USA (Michigan) 2009 [23]	25/F	Car accident with burning caused by gasoline	45	Obesity	Face	Fever, no specific lesion. Early biopsy	D9 (C)	*Mucor* sp.	L-AmB IV and local (D9) Debridement	Improved
USA (Texas) 2009 [26]	10/M	Warehouse fire	96	No	Jaw	NA	NA	NA	Mandibular debridement	Improved
Czech Republic (Brno) 2009 [36]	NA	NA	82	NA *Bact*	NA	NA	NA	*Absidia* sp., *Aspergillus fumigatus*, *Fusarium* sp.	NA	Deceased
Germany (Halle) 2010 [35]	29/M	Clothes ignition during welding. No exposure to infectious environment.	54	No *ABT*	Chest, neck, axilla, left shoulder, back	New deep muscular tissues necrosis under the skin grafts, systemic signs of sepsis	D13 (C)	*Rhizopus oryzae*	AmB IV (D13) Debridement	Deceased D15
Australia (Perth) 2010 [39]	34/M	Plane crash. Flames extinguished by muddy ground water	60	No *Bact & ABT*	Legs, left upper limb, back, abdomen	Necrosis, vesiculo-bullous rash, progressive myconecrosis	NA (H, C)	*Rhizomucor variabilis* ^£^	L-AmB IV Debridement Left above-knee and right below-knee amputations	Improved
USA (Texas) 2011 [18]	26/M	Improvised explosive device blast in Iraq	56	No *Bact*	Head and neck, retro-orbitary	Proptosis of left eye	D14 (H,C)	*Saksenaea erythrospora* **	Enucleation of the left eye	Deceased D100
China (Beijing) 2012 [31]	24/M	NA	80	No *Bact & ABT*	Thigh, chest	Dark necrotic muscle on right thigh and chest	D24 (H,C)	Mucorale	L-AmB IV (D26) Debridements	Deceased D33
USA (Chicago) 2014 [19]	20/M	Doused in gasoline and set on fire	92	No	Left upper extremity	Development of nonviable muscle tissue that demonstrated black plaques and white nodules	D13 (H,C)	Mucorale *Fusarium* sp.	AmB IVand local; L-AmB (D13) Debridement Left upper extremity amputation	Deceased D31
India (New Delhi) 2014 [30]	20/F	Accidental flame burns while cooking	60	No *Bact & ABT*	Thighs	Liquefaction and necrosis on initial deep dermal burns	D14 (H,C)	*L. ramosa*	AmB IV (D14) Debridement	Improved
USA (Texas) 2014 [22]	21/M	U.S. Marine—self-immolation with gasoline while stationed in Okinawa, Japan	90	No	Back	NA	D8 (H,C)	Apophysomyces variabilis **	L-AmB IV (D8) Debridement	Deceased
USA 2015 [21]	30/M	Improvised explosive device blast in Afghanistan	20	No	Lower extremities	NA	D3 (H,C)	*Saksenaea erythrospora, A. flavus, A. terreus, Fusarium*	L-AmB IV (D15) Debridement Lower extremities amputation	Improved
USA (Texas) 2015 [28]	22/M	Improvised explosive device blast in Afghanistan	19	No *Bact & ABT*	Upper and lower extremities, left abdomen, brain	Plant material and necrotic tissue within wounds	D17 (C)	Saksenaea vasiformis **, Cunninghamella elegans **, Lichtheimia corymbifera **, Aspergillus, Geotrichum, Alternaria	L-AmB IV (D12) and local AmB DebridementRight above-knee amputation and hip disarticulation	Deceased D44
Iran (Shiraz) 2017 [29]	50/F	NA	4–5	Diabetes mellitus	Right arm, chest, breast	Deep and extensive necrosis of muscles, systemic signs of sepsis	D17 (H)	NA	Voriconazole IV (D17) Debridement	Improved
USA (New York) 2017 [20]	56/M	Rollover motor vehicle accident (struck a tree)	20	Diabetes mellitus, hypothyroidism, hepatitis C, intravenous drug abuse *Bact & ABT*	Lower extremities	Sloughing of the grafts, necrotic skin and muscle, systemic signs of sepsis	D43 (H,C)	*Mucor* sp.	Debridement Below-knee and above-knee amputation	Deceased D44
India (New Delhi) 2018 [33]	40/M	NA	NA	No	Lower limb (osteomyelitis) and sinus	Black eschar with pale granulation; Pain, fever, chills	D150 (H)	NA	L-AmB IV, posaconazole VO Debridement Sinus tract excision; above knee amputation	Improved
India (Heydarabad) 2018 [34]	32/M	High-voltage electrical contact	NA	No	Scalp	Hyphae over wound margin and gangrenous changes over scalp surrounding the wound	D12 (C)	*Absidia corymbifera*	AmB IV Debridement	Improved
USA (Minnesota) 2019 [27]	63/M	Workplace explosion	47	Diabetes mellitus, myocardial infarction *Bact & ABT*	Upper extremities chest, abdomen, and flanks	Multiple small white plaque	D10 (C)	*Lichtheimia* sp.	Posaconazole IV (D10), isavuconazole IV (D22), AmB local Debridement	Improved

* Presence of bacterial co-infection and antibiotic treatment are indicated by “Bact” or “ABT”, respectively; ^£^ *Rhizomucor variabilis* var. *variabilis* is currently known as *Mucor irregularis*; NA = Not available; AmB = Amphotericin B, L-AmB = Liposomal Amphotericin B.
